# A Patented Rapid Method for Identification of Italian Diatom Species

**DOI:** 10.3390/ijerph16203933

**Published:** 2019-10-16

**Authors:** Camilla Puccinelli, Stefania Marcheggiani, Laura Mancini

**Affiliations:** Istituto Superiore di Sanità (ISS), Environment and Health Dept., Ecosystem and Health Unit, Viale Regina Elena 299, 00161 Rome, Italy; stefania.marcheggiani@iss.it (S.M.); laura.mancini@iss.it (L.M.)

**Keywords:** diatoms, morphological identification, flow chart

## Abstract

The study of diatoms—unicellular algae of the class Bacillariophyceae—has several applications, first and foremost the evaluation of freshwater ecosystem quality according to the Water Frame Directive 2000/60/EC (WFD). Identification at the species level is a crucial step in diatom studies, considering that species belonging to the same genus have different geographical distributions and different ecological requirements. The Rapid Method for Identification of Italian Diatom Species is aimed at guiding users in the classification of freshwater diatom species. It consists of a digitized flow chart that leads, step by step, to the identification, starting with an image capture by light or electron microscopy. This rapid and easy tool could be useful to workers of an environmental agency when performing the operational monitoring required by the WFD to classify surface waters. It will also expand the application of diatoms in numerous fields. This method has been patented in Italy.

## 1. Introduction

“Few objects are more beautiful than the minute siliceous cases of the diatomaceae: were these created that they might be examined and admired under the higher powers of the microscope?” asked Charles Darwin in 1872 [1]. 

Diatoms are unicellular algae (class Bacillariophyceae) distinguished by the presence of an inorganic cell wall composed of silica, called a frustule. They are widespread in all aquatic ecosystems, representing one of the most important and dominant components of the benthic and planktonic assemblage and contributing to about 25% of global primary production [2,3,4,5].

Diatoms also represent an economic resource: Diatomaceous Earth is a strong natural insecticide that can be a practical alternative to synthetic pesticides in some applications; their lipid contents can be used in biofuel production; the physical properties of the frustules or their nanostructure can be exploited in nano applications [6,7,8,9,10]. Furthermore, diatoms are studied in many fields [11]. Their presence in archeological sediments is considered a marker of sediment provenience and an indication of human site use. They are useful in ecological reconstructions and in paleolimnological studies [12,13]. In the forensic context, they are analyzed in organs and they can indicate where drowning has taken place in an aquatic ecosystem [14].

Diatoms are also important for the evaluation of water quality, being a key element (together with aquatic invertebrates, macrophytes and fish fauna) for the assessment of ecological status [15]. They respond to variation of nutrients and organic pollution, and evidence of eutrophication can be recorded by the alteration of their communities, i.e., a decrease of oligotrophic species and an increase of tolerant ones [16,17,18,19,20,21].

These algae can also indicate hydromorphological alterations: a shift from adnate pioneer taxa to filamentous species can be a response to a variation of current speed; degradation of river habitat is indicated by a dominance of planktonic forms instead of benthic ones [22,23,24]. Furthermore, malformation of their frustule, like an abnormal valve outline or abnormal striae pattern, can indicate the presence of chemical contaminants, taken up within the cell and inducing an alteration of the frustule [25,26]. 

Considering that at least 30,000 diatom species are estimated for all aquatic ecosystems [27], their identification is the most difficult and crucial step in diatom studies. It requires knowledge of hundreds of genera, often with variations, and frequent consultation of iconographic guides [28]. Last but not least, the main guides are in German [29,30,31,32,33,34].

Even if species belong to the same genus, they can have different geographical distributions (i.e., fresh or sea water) or have different ecological requirements [35,36]. For this reason, classification of diatoms at the species level is necessary in most applications, such as ecological status assessment methods [19,20,21,37].

Light microscopy is the most common tool for diatom identification. It is normally used by workers of environmental agencies for operational monitoring and is also required by standard procedures [38]. Scanning electron microscopy (SEM) allows analysis of the three-dimensional structure of diatom valves [39]. New methods have recently been developed for more detailed analyses of frustules, such as those merging scanning electron microscopy and digital holography microscopy or atomic force microscopy [40].

As an aid in identification, diatom image databases have been created and are available online: “Common Freshwater Diatoms of Britain and Ireland”; “Freshwater Diatom Flora of Britain and Ireland”, “Diatoms of the United States” and “Atlas of Benthic Diatoms of Italian Watercourses” [41,42,43,44]. However, none of these are an electronic guide for the identification of taxa.

Other software packages have been developed for automatic identification of diatom images. The Automatic Diatom Identification and Classification (ADIAC) project was a pilot study aimed at automatizing the identification of diatoms through image processing to analyze features such as valve shape, contour and texture of pennate diatoms [28]. The Shape Recognition Processing and Analysis (SHERPA), based on the ADIAC database, offers a versatile image processing workflow focused on the identification and measurement of diatom outlines, such as shapes and segmentation [45].

The Rapid Method for Identification of Italian Diatom Species is aimed at guiding users in the classification of freshwater diatom species. It consists of a digitized flow chart that leads, step by step, to the identification, starting with an image capture by light or electron microscopy. The method was recently patented by the Italian Patent and Trademark Office [46]. In this work, we describe the logical procedures of this method, illustrating the contents of the flow chart and its construction.

## 2. Materials and Methods 

**Figure 1:**
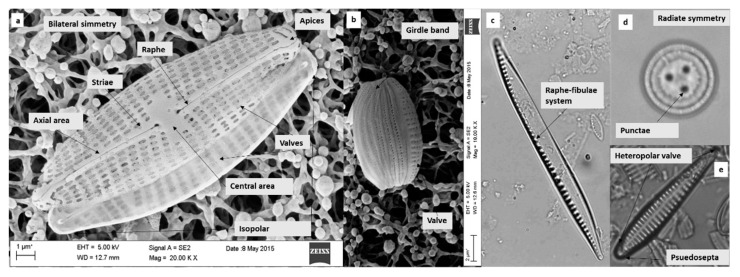
Main features of the valves visible under SEM: pennate diatom frustule in the valve view (**a**) and girdle view (**b**) with the main features of the valves; and under light microscopy: pennate diatom (**c**,**e**) and centric diatom (**d**).

Diatom identification is based on morphological observations of the frustule. It consists of two valves held together by a girdle band. Valve features, such as the shape and apices, presence of areolae, punctae or pseudosepta, the pattern and distribution of striae, can be considered species-specific (Figure 1). Light microscopy with a 100× objective [38] or SEM, with the aid of image capturing software, are needed to observe the frustule and its distinctive characters. 

### The Flow Chart—FC

The flow chart (FC) was developed on a dataset of diatoms selected from the Atlas of Benthic Diatoms of Italian Watercourses [44].

In iconographic manuals [29,30,31,32,33,34,44,47,48,49,50,51], diatom species are described based on their shape, raphe features, apices, central and axial area or fibulae and striae, as well as measurements. The distinctive characters used to construct the FC were chosen according to the contents of these descriptions:

- Symmetry and shape of the frustule;
- Absence or presence of the raphe;
- Position of the raphe;
- Polarity of valves;
- Description of apices;
- Description of the central area;
- Description of the axial area;
- Presence of fibulae or striae;
- Presence of punctae;
- Presence of pseudosepta;
- Measurements (valve length, width and number of striae or fibulae in 10 µm).

In constructing the FC, several combinations of distinctive characters were considered, since not all of them are always needed for identification at the species level. 

## 3. Results

**Figure 2:**
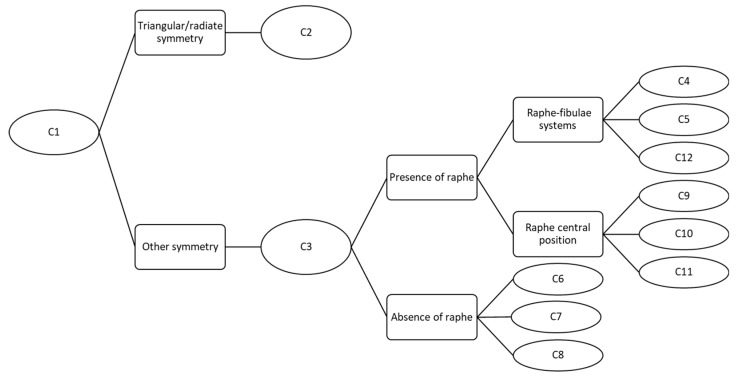
Sequence and connection of the 12 charts. Only characters that lead to each chart are reported.

The FC consists in 12 sequential charts (Figure 2) that allow the identification of 60 freshwater diatom species belonging to 25 genera widespread among different types of Italian watercourses (Appendix A).

The FC allows the user to arrive at the genus and species starting with an image and measurements. For example, for the pennate diatom in Figure 1a with “other symmetry”, the raphe in central position would lead (after C1 and C3) to one of C9, C10 or C11 according to other distinctive characters. Instead, for the centric diatom (Figure 1d), the radiate symmetry would lead to C2. The genus or family levels described in each chart are reported in Table 1.

The charts present a common last step: “confirmation of measurements”, i.e., width, breadth and number of striae. The Rapid Method for Identification of Italian Diatom Species has a dataset containing all the measurements of diatom species; confirmation of the measurements is performed by comparing observed measurements with those reported in the dataset. Whenever the measurements do not match, the process should restart from the beginning of the FC.

### 3.1. Chart C1. Melosira and Rhoicosphenia 

**Figure 3:**
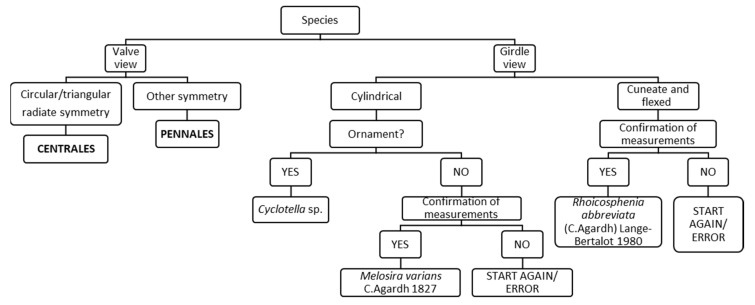
Chart C1. Introduction to the two main orders of diatoms Centrales and Pennales, and description of *Melosira varians* and *Rhoicosphenia abbreviata* in the girdle view.

In Chart C1 the FC begins with “girdle” (lateral) and “valve” (frontal) view (Figure 3). The second step regards the symmetry of the valve and identifies the two main orders Pennales or Centrales; in the latter case, the step asks about the shape of the valve (cylindrical or cuneate): for example, if valve shape is cuneate or flexed, it leads to identification of the species *Rhoicosphenia abbreviata*.

### 3.2. Chart C2. Cyclotella and Melosira 

**Figure 4:**
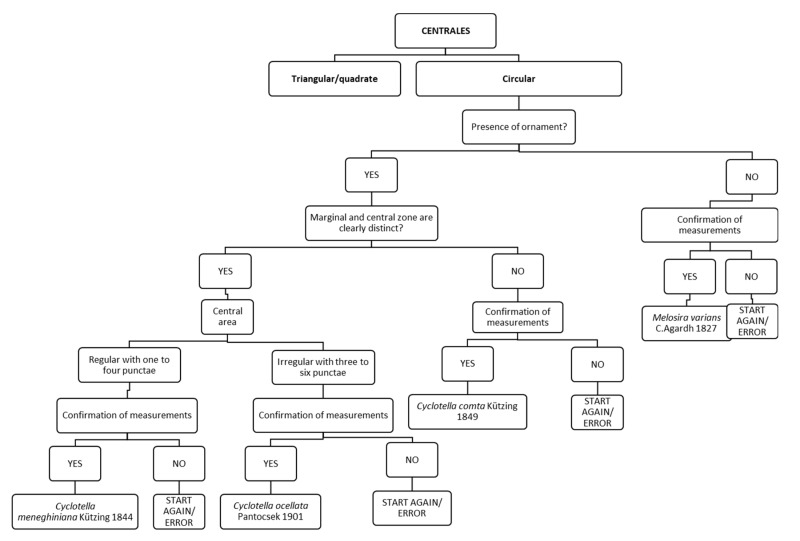
Chart C2. Description of centric diatom species such as *Cyclotella meneghiniana*, *Cyclotella ocellata*, *Cyclotella comta* and *Melosira varians*.

Chart C2 refers to the description of species of the order Centrales frequently found in rivers and belonging to *Cyclotella* and *Melosira* (Figure 4). The first step introduces the shape of the valve: triangular/quadrate or circular. After the choice of “circular shape”, the chart asks about the presence or absence of “ornament”. If present, the following step refers to the description of the central zone (if it is clearly distinct from the marginal zone) and then if it is regular or irregular with punctae. This part of the chart leads, after confirmation of measurements, to the identification of *Cyclotella meneghiniana* and *Cyclotella ocellata*. If ornament is absent, that part of the chart leads to the identification of *Melosira varians.*

### 3.3. Chart C3. Bacillariaceae, Surirellaceae and Rhopalodiaceae 

**Figure 5:**
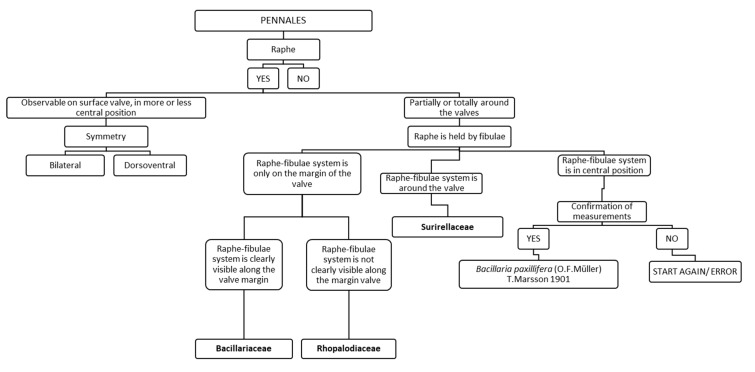
Chart C3. Description of the families Bacillariaceae, Rhopalodiaceae, Surirellaceae and of *Bacillaria paxillifera*.

Chart C3 introduces the user to the genera of Pennales (Figure 5). The description of the raphe, observable on the surface of the valve approximately in a central position or observable partially or totally around the valve and held by fibulae, leads either to a second step referring to bilateral or dorsoventral symmetry of the valve, or to a second step referring to the position of the raphe–fibulae system. After the choice of the latter, if the raphe is all around the valve the diatom is a Surirellaceae, if it is in a central position the diatom is identified as *Bacillaria paxillifera*. Instead if the raphe–fibulae system is clearly visible or not on the margin of the valve, the diatom will belong respectively to Bacillariaceae or Rhopalodiaceae.

### 3.4. Chart 4. Rhopalodia and Epithemia 

**Figure 6:**
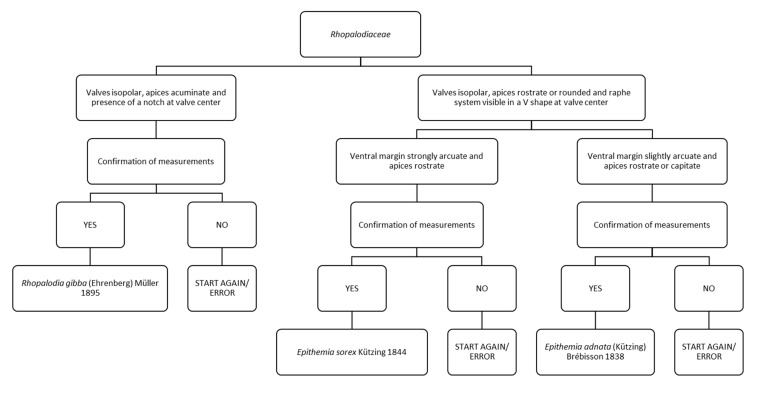
Chart C4. Description of species of Rhopalodiaceae: *Rhopalodia gibba, Epithemia sorex* and *Epithemia adnata*.

Chart C4 describes three species of Rhopalodiaceae (Figure 6). The first step describes “valves isopolar, apices acuminate and presence of a notch at valve center” or “valves isopolar, apices rostrate or rounded and raphe–fibulae system visible in a V shape at valve center”. The former leads to *Rhopalodia gibba*. The latter leads to a step referring to ventral valve margin strongly arcuate and apices rostrate or to ventral margin slightly arcuate and apices rostrate or capitate. In the first case it leads to the identification of *Epithemia sorex*, in the second case to *Epithemia adnata.*

### 3.5. Chart C5. Nitzschia 

**Figure 7:**
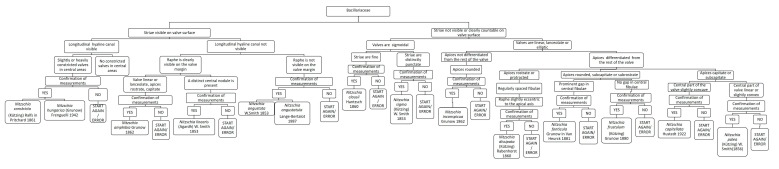
Chart C5 *Nitzschia* genus description. On the left, when striae are visible on the valve surface. Description of *Nitzschia constricta*, *N. hungarica*, *N. amphibia*, *Nitzschia* *linearis*, *Nitzschia* *angusta* and *Nitzschia* *angustatula*. On the right, when striae are not visible on the valve surface. Description of *Nitzschia* *clausii, Nitzschia* *sigma, Nitzschia* *inconspicua, Nitzschia* *dissipata*, *Nitzschia* *fonticola, Nitzschia* *frustulum, Nitzschia* *capitellata* and *Nitzschia* *palea*.

Chart C5 describes species of the genus *Nitzschia* (family Bacillariaceae; Figure 7). In the first step, it asks if striae are clearly visible or not on the valve surface. When striae are clearly visible, if a longitudinal hyaline canal is present and then the valves are slightly or heavily constricted it leads to the identification of *Nitzschia constricta*, after confirmation of measures (Figure 7). When there is no longitudinal hyaline canal, the following step asks if the raphe is clearly visible on the valve margin. If yes and a central node is present, it leads to the identification of *Nitzschia linearis*. If there is not a central node but the valves are linear or lanceolate and apices rostrate, it leads to *Nitzschia amphibia*. If the raphe is not visible on the valve, measurements can represent the main factor for identification of *Nitzschia angustata* or *Nitzschia angustatula*.

If in the first steps, striae are not visible, if the raphe system is slightly eccentric to the apices of the valves and fibulae are regularly spaced, the diatom is identified as *Nitzschia dissipata* (Figure 7). Instead, if striae are not clearly countable, it asks if the shape of the valves is sigmoidal, linear or lanceolate. If sigmoidal and the striae are fine, it leads to *Nitzschia clausii*; if the striae are coarse, it leads to *Nitzschia sigma*. If the valves are linear and the apices are not differentiated from the rest of the valve, it leads to *Nitzschia inconspicua*.

### 3.6. Chart C6. Rhoicosphenia, Fragilaria, Ulnaria, Staurosira and Pseudostaurosira 

**Figure 8:**
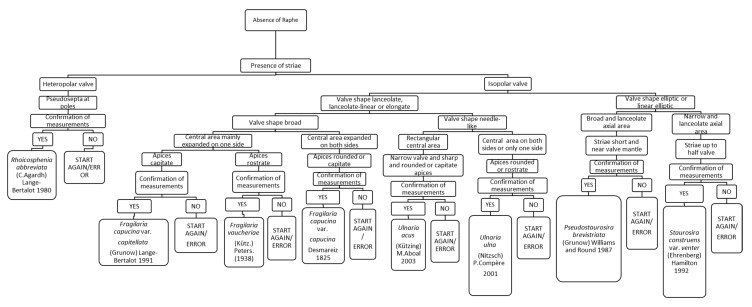
Chart C6. Diatoms without raphe and with striae. Description of *R. abbreviata, Fragilaria capucina* var. *capitellata, Fragilaria vaucheriae, Fragilaria capucina* var. *capucina, Ulnaria acus, Ulnaria ulna, Pseudostaurosira brevistriata* and *Staurosira construens* var. *venter*.

Chart C6 describes diatoms without raphe and with the presence of striae (Figure 8). The first step concerns the polarity of the valve. For example, if it is heteropolar and there are pseudosepta at the poles, the diatom is *Rhoicosphenia abbreviata*. If the valve is isopolar and linear or lanceolate, the following step asks about the description of the central area: if it is expanded on only one side of the valve or on both sides. In the former case, if the apices are capitate, the species is *Fragilaria capucina*, whereas if the apices are rostrate, the species is *Fragilaria vaucheriae*.

### 3.7. Chart C7. Planothidium, Achnanthidium and Cocconeis 

**Figure 9:**
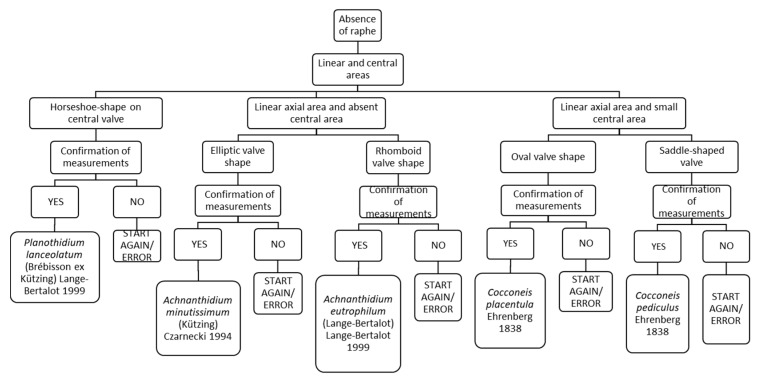
Chart C7. Diatoms without raphe and with an irregular area. Description of *Planothidium lanceolatum, Achnanthidium minutissimum, Achnanthidium eutrophilum, Cocconeis placentula* and *Cocconeis pediculus*.

Chart C7 refers to diatoms without raphe and to the description of the axial and central areas (Figure 9). For example, “Horseshoe shape on central valve” leads to *Planothidium lanceolatum*, while “Linear axial area and absent central area” and elliptic valve shape leads to *Achnanthidium minutissimum*. Instead, if there is a linear axial area and small central area, an oval-shaped valve leads to *Cocconeis placentula*.

### 3.8. Chart C8. Diatoma and Meridion 

**Figure 10:**
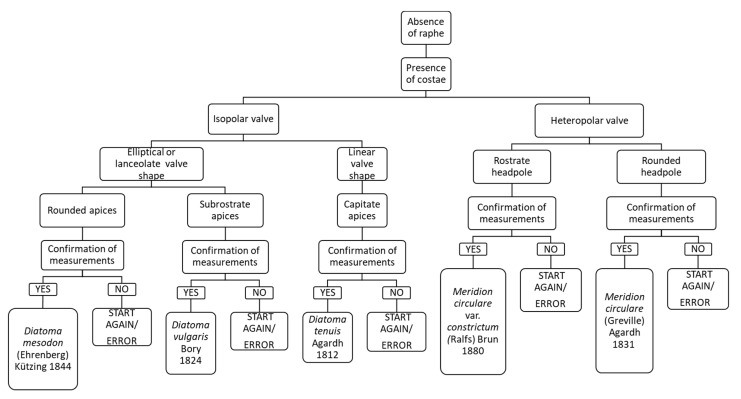
Chart C8. Diatoms without raphe and with costae. Description of *Diatoma mesodon, D. vulgaris, Meridion circulare* var. *constricta* and *Meridion circulare*.

Chart C8 describes Araphidae species that present costae (Figure 10). The following step refers to an isopolar or heteropolar valve: if the valve is heteropolar and the headpole is rounded, it leads to the identification of *Meridion circulare*; if the valve is isopolar, with linear shape and apices capitate, it leads to *Diatoma tenuis*.

### 3.9. Chart C9. Achnanthidium, Eolimna, Sellaphora, Navicula and Cocconeis 

**Figure 11:**
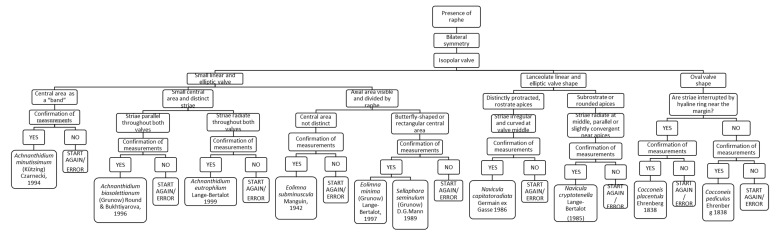
Chart C9. Diatoms with bilateral symmetry and raphe. Description of *A. minutissimum, A. biasolettianum, A. eutrophilum, Eolimna subminuscula, Eolimna minima, Sellaphora seminulum, Navicula capitatoradiata, Navicula cryptotenella, Cocconeis placentula* and *Cocconeis pediculus.*

Chart C9 refers to diatoms with a valve with raphe with bilateral symmetry—Monoraphidae and Biraphidae (Figure 11). The steps describe the shape and dimensions of the valves, and then the characteristics of the striae and central area. For instance, a species with a small linear valve and a central area as a “band” is *Achnanthidium minutissimum*. Instead, if the valve has an oval shape, the following step asks if the “Striae are interrupted by hyaline ring near the margin”: if yes, it leads to the identification of *Cocconeis placentula*; if the striae are not interrupted, it leads to the identification of *Cocconeis pediculus*.

### 3.10. Chart C10. Gomphonema and Rhoicosphenia 

**Figure 12:**
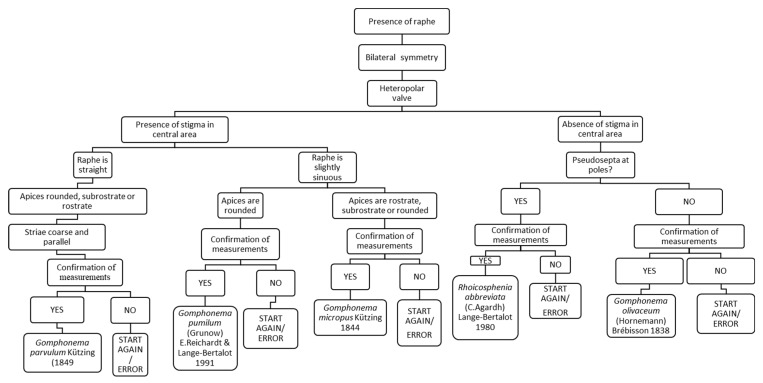
Chart C10. Diatoms with a heteropolar valve, with presence or absence of a stigma in the central area. Description of *Gomphonema parvulum, Gomphonema pumilum, Gomphonema micropus, Rhoicosphenia abbreviata* and *Gomphonema olivaceum*.

Chart C10 describes a heteropolar valve with raphe, with species belonging to the genera *Gomphonema* and *Rhoicosphenia* (Figure 12). For example, the first question refers to the presence or absence of a stigma: if absent, and then there are not pseudosepta, it leads to the identification of *Gomphonema olivaceum*; instead, if pseudosepta are present, the species is *Rhoicosphenia abbreviata*.

### 3.11. Chart C11. Amphora and Cymbella 

**Figure 13:**
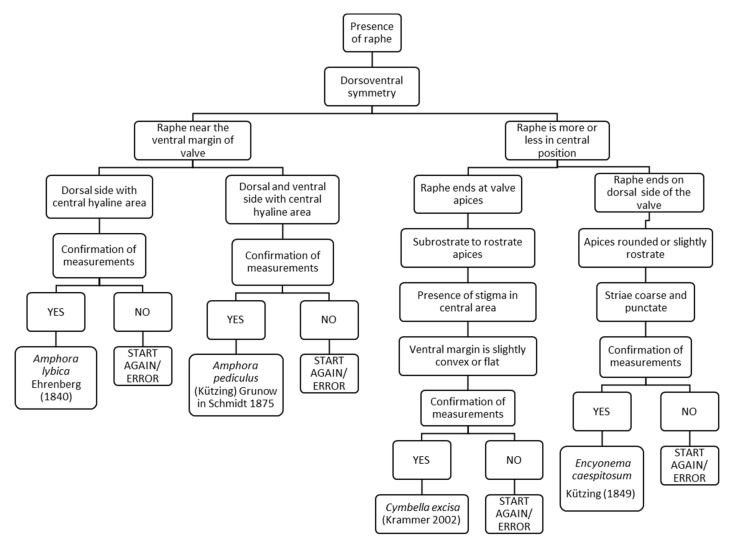
Chart C11. Diatoms with dorsoventral symmetry, with raphe near the ventral margin or in a central position. Description of *Amphora lybica, Amphora pediculus, Cymbella excisa* and *Encyonema caespitosum.*

Chart C11 refers to a valve with raphe with dorsoventral symmetry (Figure 13). The following steps ask about the position of the raphe and if it ends at the apices of the valve or on the dorsal side of the valve. For example, if the raphe is near the ventral margin of the valve and the dorsal side has a central hyaline area, it leads to *Amphora lybica*. Instead, if the raphe is more or less in a central position, ends on the dorsal side of the valve and the apices are rounded or slightly rostrate, it leads to *Encyonema caespitosum*.

### 3.12. Chart C12. Cymatopleura and Surirella 

**Figure 14:**
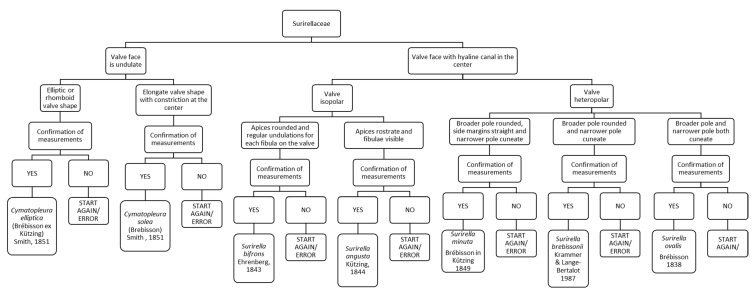
Chart C12. Description of Surirellaceae, when the raphe is all around the valve: *Cymatopleura elliptica, Cymatopleura solea, Surirella bifrons, Surirella angusta, Surirella minuta, Surirella brebissonii* and *Surirella ovalis.*

Chart C12 refers to diatoms presenting the raphe all around the valve (Surirellaceae; Figure 14). The first step refers to the valve face, with the presence of ornaments like undulations or a hyaline canal. If the valve face is undulate and the valve is elliptic or rhomboid, it leads to *Cymatopleura elliptica*. Instead, if there is a hyaline canal and the valve is isopolar and apices are rostrate and fibulae visible, it leads to *Surirella angusta*. 

### 3.13. Some Applications of the Flow Chart

**Figure 15:**
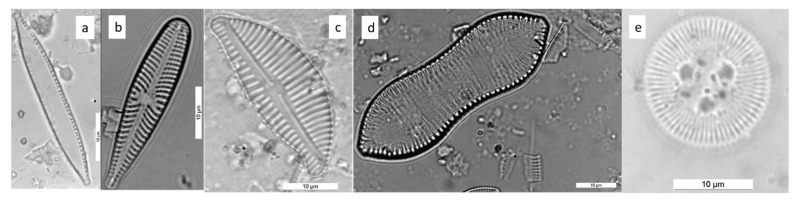
(**a**) *Nitzschia capitellata*, (**b**) *Gomphonema olivaceum*, (**c**) *Encyonema caespitosum*, (**d**) *Cymatopleura solea* and (**e**) *Cyclotella ocellata*.

In this section, we reported some examples of diatom identification using the flow chart. Figure 15 shows five diatoms of different genera. 

Figure 15a shows a valve with “other symmetry” (Chart C1 to Chart C3); the raphe is present, partially around the valve, and has a system with fibulae. The raphe–fibulae system is only on the margin of the valve and clearly visible. This diatom belongs to Bacillariaceae (Chart C3 to Chart C5). Striae are not clearly visible (Figure 7), valves are lineate, apices are differentiated from the rest of the valve and capitate or subrostrate, and the central part of the valve is slightly convex. After confirmation of the measurements, this diatom can be identified as *N. capitellata*.

Figure 15b is a valve with “other symmetry” (Chart C1 to Chart C3); the raphe is present in a central position and the valve is heteropolar (Chart C3 to Chart 10). There is no stigma in the central area and there are no pseudosepta at the poles. After confirmation of the measurements, this diatom can be identified as *G. olivaceum*.

Figure 15c is a valve with “other symmetry” (Chart C1 to Chart C3); the raphe is present in a central position and the symmetry is dorsoventral (Chart C3 to Chart C11). The raphe is more or less at the center of the valve and ends on the dorsal side of the valve. The apices are rostrate and striae are coarse and punctate. After confirmation of the measurements, this diatom can be identified as *E. caespitosum*.

Figure 15d c is a valve with “other symmetry” (Chart C1 to Chart C3). The raphe is present all or partially around the valve and held by fibulae. In this case, the raphe-fibulae system is all around the valve and this diatom belongs to Surirellaceae (Chart C3 to Chart C12). The valve has an undulate face and elongate shape with a constriction at the center. After confirmation of the measurements, this diatom can be identified as *C. solea.*

Figure 15e is a valve with radiate/triangular symmetry (Chart C1 to Chart C2). This valve has circular symmetry with a clear distinction between marginal and central area. The latter is irregular and presents 3 punctae. After confirmation of the measurements, this diatom can be identified as *C. ocellata*.

## 4. Discussion 

Diatom identification is the main source of errors in the entire process of diatom analysis [52,53], and it also requires much time to perform. This is due to the great number of species, lack of experience of the person carrying out the identification, and also the fact that the most important iconographic guides are in German [29,30,31,32,33,34]. The use of the flow chart reduces this source of error because it closely guides the user to reach the name of the species. The FC focuses on distinctive characters of each species, reducing the time of searching for the diatom in different manuals.

Dichotomous keys of diatoms usually follow the taxonomic hierarchy of Bacillariophyceae: they describe Centrales (radiate symmetry) separately from Pennales (bilateral symmetry), and Araphidae (absence of raphe) from Monoraphidae (presence of raphe on only one valve) or Biraphidae (presence of raphe on the two valves) [44,51]. Instead, the FC is based on the analysis of a diatom image, and it has been structured to be able to describe several possible images. For example, it also makes it possible to analyze a girdle view of a diatom: only a few diatom species can be surely identified by a girdle view, such as *Melosira varians* and *Rhoicosphenia abbreviata* (Figure 3). Monoraphidae species such as *Achnanthidium minutissimum* and *Cocconeis pediculus* have been described in separate charts (C7 and C9), the two possible valves without and with raphe. Furthermore, one species, *Rhoicosphenia abbreviata* is described in a girdle view and in a valve view with or without raphe (Figure 3, Figure 8, Figure 12).

It is essential to have the measurements of the diatom to obtain the classification of the species: if the measurements taken by the operators match those included in the software, a species name will be attributed to the image; if not, the identification process will start over from the beginning. 

At present, this method allows the identification of 60 freshwater diatoms. The species were selected based on their distribution along Italian watercourses and on their sensitivity to water quality; for instance, *A. minutissimum* and *D. mesodon* are typical of oligotrophic rivers, *M. varians* and *N. capitatoradiata* are found in mesotrophic waters, and *E. minima* and *N. hungarica* are characteristic of eutrophic waters [16,44].

Due to its structure, the FC can be supplemented with further species, with the inclusion of new descriptions or even new charts: for example, in Chart C2 (Figure 4), new Centrales species could be added, e.g., of the genus *Stephanodiscus*, or in Chart C9 the identification of the genus *Gyrosigma* could be added, with the description of the sigmoidal shape of the valve (Figure 11). Furthermore, considering the importance of analyses of teratological forms in order to detect contamination by heavy metals or pesticides, new charts could contain the description of abnormal valve shape or abnormal patterns of striae.

Other software like ADIAC or SHERPA is aimed at making identification completely computerized through the segmentation of each diatom image, based on a large database of light microscopy images. Instead, the Rapid Method for Identification of Italian Diatom Species is based on a flow chart including 11 distinctive characters, which guides the user in the identification process. The FC can be applied to different images of diatoms performed by light microscopy, also at different magnifications (40× and 100×), or by electron microscopy.

## 5. Conclusions

In conclusion the identification process (the FC) is rapid and user friendly. The Rapid Method for Identification diatom at species level, which is currently calibrated on species found in the river typologies present in Italian territory, can be useful in ecological studies of other countries improving the species dataset. It can also be used for other applications such as investigations of diatoms in drowning cases, archeological and paleolimnological studies.

## 6. Patents

The Rapid Method for Identification of Italian Diatom Species was granted an Italian patent no. 102017000007096 on 4/06/2019.

## Figures and Tables

**Table 1:** The 12 charts forming the flow chart (FC) and the genera or families (* family level) described.

Chart Code	Genera
C1	*Melosira* and *Rhoicosphenia*
C2	*Cyclotella* and *Melosira*
C3	Bacillariaceae, Surirellaceae, Rhopalodiaceae *
C4	*Rhopalodia*, *Epithemia*
C5	*Nitzschia*
C6	*Rhoicosphenia*, *Fragilaria*, *Ulnaria*, *Staurosira*, *Pseudostaurosira*
C7	*Planothidium*, *Achnanthidium*, *Cocconeis*
C8	*Diatoma* and *Meridion*
C9	*Achnanthidium*, *Eolimna*, *Sellaphora*, *Navicula*, *Cocconeis*
C10	*Gomphonema*, *Rhoicosphenia*
C11	*Amphora*, *Cymbella*
C12	*Cymatopleura* and *Surirella*

## Appendix A

**Table A1:** List of diatom species included in the Rapid Method for Identification of Italian Diatom Species.

Diatom Species
*Achnanthidium biasolettianum* (Grunow) Round and Bukhtiyarova, 1996
*Achnanthidium eutrophilum* (Lange-Bertalot) Lange-Bertalot 1999
*Achnanthidium minutissimum* (Kützing) Czarnecki 1994
*Amphora lybica* Ehrenberg (1840)
*Amphora pediculus* (Kützing) Grunow in Schmidt 1875
*Bacillaria paxillifera* (O.F. Müller) T. Marsson 1901
*Cocconeis pediculus* Ehrenberg 1838
*Cocconeis placentula* Ehrenberg 1838
*Cyclotella comta* Kützing 1849
*Cyclotella meneghiniana* Kützing 1844
*Cyclotella ocellata* Pantocsek 1901
*Cymatopleura elliptica* (Brébisson ex Kützing) Smith, 1851
*Cymatopleura solea* (Brébisson) Smith, 1851
*Cymbella excisa* (Krammer 2002)
*Diatoma mesodon* (Ehrenberg) Kützing 1844
*Diatoma tenuis* Agardh 1812
*Diatoma vulgaris* Bory 1824
*Encyonema caespitosum* Kützing (1849)
*Eolimna minima* (Grunow) Lange-Bertalot, 1997
*Eolimna subminuscula* Manguin, 1942
*Epithemia sorex* Kützing 1844
*Epithemia adnata* (Kützing) Brébisson 1838
*Fragilaria capucina* var. *capucina* Desmareiz 1825
*Fragilaria capucina* var. *capitellata* (Grunow) Lange-Bertalot 1991
*Fragilaria vaucheriae* (Kütz.) Peters. (1938)
*Gomphonema olivaceum* (Hornemann) Brébisson 1838
*Gomphonema parvulum* Kützing (1849)
*Gomphonema pumilum* (Grunow) E. Reichardt and Lange-Bertalot 1991
*Gomphonema micropus* Kützing 1844
*Melosira varians* C. Agardh 1827
*Meridion circulare* (Greville) Agardh 1831
*Meridion circulare* var. *constrictum* (Ralfs) Brun 1880
*Navicula capitatoradiata* Germain ex Gasse 1986
*Navicula cryptotenella* Lange-Bertalot 1985
*Nitzschia fonticola* Grunow in Van Heurck 1881
*Nitzschia hungarica* (Grunow) Frenguelli 1942
*Nitzschia amphibia* Grunow 1862
*Nitzschia angustata* W. Smith 1853
*Nitzschia angustatula* Lange-Bertalot 1987
*Nitzschia capitellata* Hustedt 1922
*Nitzschia clausii* Hantzsch 1860
*Nitzschia constricta* (Kützing) Ralfs in Pritchard 1861
*Nitzschia dissipata* (Kützing) Rabenhorst
*Nitzschia frustulum* (Kützing) Grunow 1880
*Nitzschia linearis* (Agardh) W. Smith 1853
*Nitzschia palea* (Kützing) W. Smith 1856
*Nitzschia sigma* (Kützing) W. Smith 1853
*Planothidium lanceolatum* (Brébisson ex Kützing) Lange-Bertalot 1999
*Pseudostaurosira brevistriata* (Grunow) Williams and Round 1987
*Rhoicosphenia abbreviata* (C. Agardh) Lange-Bertalot 1980
*Rhopalodia gibba* (Ehrenberg) Müller 1895
*Sellaphora seminulum* (Grunow) D.G. Mann 1989
*Staurosira construens* var. *venter* (Ehrenberg) Hamilton 1992
*Surirella angusta* Kützing, 1844
*Surirella bifrons* Ehrenberg, 1843
*Surirella brebissonii* Krammer and Lange-Bertalot 1987
*Surirella minuta* Brébisson in Kützing 1849
*Surirella ovalis* Brébisson 1838
*Ulnaria acus* (Kützing) M. Aboal 2003
*Ulnaria ulna* (Nitzsch) P. Compère 2001
